# Value at Risk long memory volatility models with heavy-tailed distributions for cryptocurrencies

**DOI:** 10.3389/fams.2025.1567626

**Published:** 2025-05-19

**Authors:** Stephanie Danielle Subramoney, Knowledge Chinhamu, Retius Chifurira

**Affiliations:** School of Mathematics, Statistics and Computer Science, University of KwaZulu-Natal, Durban, South Africa

**Keywords:** cryptocurrency, Generalized Autoregressive Conditional Heteroskedasticity (GARCH), generalized autoregressive score (GAS), long memory (LM), Value-at-Risk (VaR)

## Abstract

This paper investigates the volatility dynamics and underlying long memory features of four major cryptocurrencies—Bitcoin, Ethereum, Litecoin, and Ripple—which were selected due to their high liquidity, large trading volumes, and historical significance in the digital asset market. The long-range dependence exhibited in cryptocurrency markets is often overlooked. However, based on the strong evidence of persistent dependence in the return series, we adopt advanced volatility models that are capable of accommodating high volatility and heavy-tails, as well as the long memory properties of cryptocurrencies. Specifically, we employ long-memory extensions of the GAS (Long memory GAS) and GARCH (Fractionally Integrated Asymmetric Power ARCH) models, integrating heavy-tailed innovation distributions: the Generalized Hyperbolic Distribution (GHD) and Generalized Lambda Distribution (GLD). Standard GARCH and GAS models are included as benchmarks. The performance of the models are assessed using Value-at-Risk (VaR) estimation, backtesting (in-sample and out-of-sample) and volatility forecasting metrics. The results indicate that long memory models, particularly the FIAPARCH model, consistently outperforms the standard GAS and GARCH models in capturing tail risk and the volatility persistence. These findings emphasize the critical role of long memory in modeling the risk of cryptocurrencies, indicating that accounting for volatility persistence can significantly enhance the accuracy of risk estimates and strengthen risk management practices.

## 1 Introduction

Long memory is a phenomenon that can be described as the persistence of volatility, suggesting that past observations have an impact on future values. In financial markets, this behavior is often exhibited in volatility and thus has crucial implications for forecasting and risk management. The long memory properties of financial assets have been substantially investigated and studied, with evidence indicating that volatility is indeed a long memory process [1–4].

Cryptocurrencies share numerous commonalities with traditional financial assets; however, the volatility dynamics of cryptocurrencies are often found to be higher than traditional assets like stocks [5] as well as leading fiat currencies [6]. The cryptocurrency market is decentralized with no association to a higher authority and thus making the market structure unique. The significant development of cryptocurrencies demonstrated by their growing transaction volume and market capitalization has distinguished cryptocurrencies as a revolutionary instrument in financial markets, internationally [7]. Specifically, the cryptocurrency market has grown significantly over the last decade or so, as the global market capitalization of cryptocurrencies surged from under 20 billion USD in early 2017 to over 2.5 trillion USD at its peak in late 2021, with average daily trading volumes increasing by more than 1,000% during this period [8]. In comparison to other emerging markets, this explosive growth and extreme price fluctuations have introduced new challenges for financial modeling. Additionally, their decentralized structure, sensitivity to sentiment, speculative trading behavior and continuous 24/7 trading with no market closures, contribute to the significant volatility persistence and clustering, suggesting that the long memory property has become particularly relevant for cryptocurrency modeling.

The volatility exhibited by cryptocurrencies has been studied extensively [9–13] and it is apparent that the market is highly volatile in nature. This extremity may result in distinct trends in volatility persistence and thus it is imperative to study if there are long memory influences in the volatility of these markets similar to that of other financial time series. Rambaccussing and Mazibas [14] investigated the long memory properties in the returns and volatility of five cryptocurrencies, Bitcoin, Litecoin, Ethereum, Bitcoin Cash, and Ripple of which they discovered that long memory may not explicitly be present in the returns of the cryptocurrencies, except for that of Ethereum where long memory does exist, however, long memory appears to be well prominent in the volatility of these cryptocurrencies. Jiang et al. [15] studied the impact of the dual long memory and structural break features of six highly-traded cryptorcurrencies. It was found that these cryptocurrencies does, in fact, exhibit both structural breaks and long memory properties in their returns, and are especially present in the volatility. Soylu et al. [7] explored the long memory traits of Bitcoin, Ethereum, and Ripple. The cryptocurrencies were tested for long memory using Rescaled Range Statistics (R/S), Gaussian Semi Parametric (GSP), and the Geweke and Porter-Hudak (GPH) Model Method. The squared returns of all three cryptocurrencies exhibited strong persistence indicating the presence of long memory.

Since long term dependencies of volatility exist in cryptocurrencies, the adoption of an appropriate model which has the capability to adequately capture the long memory traits, as well as the extreme volatility exhibited, is of significant importance to analyze and forecast the risks associated with these cryptocurrencies. The Generalized Autoregressive Conditional Heteroskedasticity (GARCH) model introduced by Bollerslev [16] is a conventional volatility model which can be extended to incorporate fractionally integrated models to encapsulate long memory properties. Although the use of the standard GARCH model is commonly employed for modeling volatility and long-memory properties, Davidson [17] established that this model may not be entirely ideal, as GARCH models do not adequately cater for outliers and thus may generate biased estimates. The effect of innovations on the subsequent conditional variance for a long memory process is generally that of a hyperbolic decay, and therefore the impact of outliers may be magnified resulting in a rising biased long memory estimate [18]. The Generalized Autoregressive Score (GAS) model, introduced by Creal et al. [19], is another volatility model that caters for the downfall of the GARCH model discussed above due to its unique robustness characteristics. The GAS model framework can also be enhanced to accommodate the presence of long memory in returns [20], making it a viable candidate to model cryptocurrencies. Chkili [21] investigated models which would best fit the long memory present in the volatility dynamics of the Bitcoin returns for the 2013–2020 period of which the Fractionally Integrated GARCH (FIGARCH) model was found to be the most favorable model. Gao and Shi [18] studied the long memory and regime switching in the second comment using GAS models. The robustness against outliers of the long memory GAS (LMGAS) model and the Markov switching GAS (MS-GAS) model was investigated utilizing West Texas Intermediate crude oil spot returns. The findings indicate that the GAS estimators were more robust when compared to its GARCH counterparts when catering for outliers. However, the LMGAS model still produced spurious long memory when a regular regime-switching process is fitted and thus an MS-LMGAS model was proposed which catered for both the spurious long memory and robustness against outliers.

Although some studies have tested long memory GARCH and GAS-type models for individual cryptocurrencies, comprehensive evaluations remain limited. In particular, few studies jointly apply these models across multiple cryptocurrencies while incorporating flexible innovation distributions. This paper aims to contribute to the literature in three key aspects. First, we explore the presence and strength of long memory features of four well established cryptocurrencies, Bitcoin, Ethereum, Litecoin, and Ripple. These cryptocurrencies were selected due to their high liquidity, large trading volumes, and their ability to represent a diverse variety of the cryptocurrency market in terms of history, market capitalization and investor profiles. Bitcoin and Ethereum dominate the market as the most capitalized and widely adopted coins whereas Litecoin and Ripple offer different technical foundations and transaction architectures which makes these cryptocurrencies collectively a robust and representative sample for assessing market behavior and long memory dynamics. Second, we conduct a comparative analysis using two long memory models, FIAPARCH and LMGAS, with standard GARCH and GAS-type models incorporating both the Generalized Hyperbolic Distribution (GHD) and the Generalized Lambda Distribution (GLD) as innovation distributions. The central aim of this study is to evaluate whether the inclusion of long memory structures and flexible distributional assumptions significantly enhances volatility modeling and risk estimation in cryptocurrency markets and thus, this analysis provides an empirical contribution by jointly modeling long memory and heavy-tailed behavior across a diverse set of cryptocurrencies which is an area of research that is not extensively explored. Third, we evaluate the performance of the models through a standard statistical fit and also through a risk perspective in that we conduct Value-at-Risk (VaR) estimation, in-sample and out-of-sample backtesting and volatility forecast accuracy. This approach allows us to evaluate the models’ ability to capture tail risk and long memory in risk management frameworks.

Our findings provide valuable insights for investors, risk managers, regulators, and academics on how long memory dynamics in different cryptocurrencies can improve their strategies to monitor and manage financial risk.

## 2 Methodology

In order to adequately model the persistent volatility dynamics and extreme behavior of cryptocurrencies, this study employs long memory volatility models. Standard GARCH and GAS-type models tend to fall short when it comes to effectively capturing both the extreme and the long term dependencies of volatility, justifying the applicability of the long memory extensions of the models in this paper (LMGAS and FIAPARCH). Cryptocurrencies, in line with other financial assets, also exhibits significant heavy-tails and skewness. Thus, heavy-tailed distributions are used for modeling the returns innovations to better facilitate flexibility and capture any underlying kurtosis and asymmetry.

The aim of this section is to outline the modeling framework used to capture the volatility and risk dynamics of the four major cryptocurrencies, Bitcoin, Ethereum, Litecoin, and Ripple. Specifically, we aim to incorporate long memory features into volatility modeling using FIAPARCH and LMGAS processes, and thereafter account for heavy-tailed behavior in return innovations through the use of a generalized hyperbolic distribution (GHD) and/or a generalized lambda distribution (GLD). We also detail how these models are evaluated using Value-at-Risk (VaR) and backtesting procedures.

This section is structured such that we first introduce the return framework used throughout this study, followed by a description of long memory processes. We then discuss the GARCH, FIAPARCH, GAS, and LMGAS models used to model the cryptocurrency returns. Finally, we introduce the heavy-tailed distributions used for the innovation distributions and then conclude with the approach used for VaR estimation and backtesting.

### 2.1 Model framework

Let $r_{t}$ represent the return at time t, modeled as,

(1) $$r_{t}=\mu_{t}+a_{t},\;a_{t}=\sigma_{t}\epsilon_{t},\;\epsilon_{t}∼D(0,1)$$

where $\mu_{t}$ is the conditional mean, $a_{t}$ is the innovation term, $\sigma_{t}^{2}$ is the conditional variance, and D(0,1) denotes a standardized distribution such as the GHD or GLD. Throughout this paper, we assume $\mu_{t}$ is constant and focus primarily on modeling $\sigma_{t}^{2}$ or related volatility dynamics.

### 2.2 Long memory processes

$X_{t}$, a weakly stationary time series, is considered an LM process if the $\tau^{th}$ autocorrelation function (ACF), denoted by ρ(τ) follows:

(2) $$\rho (\tau )\propto \tau^{2d-1},\;\text{as}\;\tau \to \infty$$

for d∈(0,0.5), where d is the long-range dependence parameter [22]. This implies the ACF exhibits a slow hyperbolic decay suggesting that dependencies persists for longer lags as opposed to a short memory process where the ACF diminishes exponentially. The rate of growth variances of partial sums can be defined as,

(3) $$var\left(\sum_{t=1}^{T}X_{t}\right)=O\left(T^{2d+1}\right)$$

where T is the time period under consideration [23].

In the following subsections we discuss how the standard GARCH and GAS models have been adjusted and developed to incorporate long memory which results in the FIAPARCH and LMGAS models.

#### 2.2.1 GARCH and FIAPARCH models

The Generalized Autoregressive Conditional Heteroskedasticity (GARCH) model, proposed by Bollerslev [16], is a widely used model for capturing volatility exhibited in financial time series.

The standard GARCH(p,q) model for $\sigma_{t}^{2}$ is,

(4) $$\sigma_{t}^{2}=\omega +\sum_{i=1}^{p}\alpha_{i}a_{t-i}^{2}+\sum_{j=1}^{q}\beta_{j}\sigma_{t-j}^{2}$$

where $a_{t}=\sigma_{t}\epsilon_{t}$ with $\epsilon_{t}$ being a sequence of *i.i.d* random variables, $\alpha_{0}>0$, $\alpha_{i}\geq 0$, $\beta_{i}\geq 0$ and $\sum_{i=1}^{max(m,s)}(\alpha_{i}+\beta_{i})<1$. $a_{t}=r_{t}-\mu_{t}$ is the mean corrected returns where $\mu_{t}$ is the mean of the return series.

A drawback of the GARCH model is that it assumes that conditional variance is linearly related to past squared returns and past variances which does not allow the model to account for asymmetric volatility or long memory properties. Thus an extension of the GARCH model, the fractionally integrated asymmetric power ARCH (FIAPARCH) model, introduced by Tse [24], permits for an asymmetric response of volatility to both positive and negative shocks, long range volatility dependence as well as the ability to allow the power of returns to be determinable by the data. A basic FIAPARCH (1, *d*, 1) model is defined as,

(5) $$(1-\phi L){\left(1-L\right)}^{d}f\left(a_{t}\right)=\alpha_{0}+[1-\beta (L)]a_{t}$$

where:

- $\beta (L)=\sum \beta_{j}L^{j}$ is the short-run lag polynomial capturing persistence, where the $\beta_{j}$ coefficients are analogous in interpretation to those in the standard GARCH model, but appear within a fractional differencing framework.
- $f\left(a_{t}\right)={\left(\left|a_{t}\right|-\gamma a_{t}\right)}^{\delta }$.
- γ is the leverage parameter, where −1<γ<1.
- δ is the parameter for the power term, where 0<δ<2.
- |ϕ| is the autoregressive parameter, where |ϕ|<1.
- $\alpha_{0}>0$.
- d is the fractional differencing parameter, where 0≤d≤1.
- L is the lag operator.

This model has the capability to capture the volatile nature of cryptocurrencies while taking into account their long memory features. The FIAPARCH process was implemented in this analysis using the **G@RCH** [25] package in the econometric software, **OxMetrics** [26].

### 2.2.2 GAS and LMGAS models

The set of generalized autoregressive score (GAS) models proposed by Creal et al. [19] differs from the conventional observation-driven models in that it adopts a scaled score of the likelihood function as the key driving factor which allows for a framework that easily introduces time-varying parameters across a range of non-linear models. Let $r_{t}$ denote the observed log-return at time t, which is the variable modeled in the GAS framework i.e., $r_{t}∼D\left(0,\sigma_{t}^{2}\right)$, with time-varying volatility driven by the GAS recursion.

The general expression of the GAS (p,q) process is defined as follows:

(6) $$f_{t+1}=\omega +\sum_{i=1}^{p}\alpha_{i}s_{t-i+1}+\sum_{j=1}^{q}\beta_{j}f_{t-j+1}$$

where $f_{t}$ is a vector of time-varying parameters at time t, ω is a vector of constants, $s_{t}$ is the scaled score of the log-likelihood function, and $\alpha_{i}$, $\beta_{j}$ are parameter matrices governing the impact of past scores and past values of $f_{t}$, respectively. Furthermore, it is important to note that $f_{t+1}$ is influenced by all initial values, θ, which is a vector of static parameters of the model. The distinguishing feature of the GAS model lies in the local score function, $\nabla_{t}$, which is given by,

(7) $$\nabla_{t}=\frac{\partial \ln p\left(r_{t}|f_{t},F_{t-1};\theta \right)}{\partial f_{t}},$$

and the scaled conditional score $s_{t}$ is of the form:

(8) $$s_{t}=S_{t}\cdot \nabla_{t}$$

where $p\left(r_{t}|f_{t},F_{t-1};\theta \right)$ is the conditional likelihood function of $r_{t}$ and $S_{t}$ is the scaling term. Various specifications of $S_{t}$ results in different models, for e.g., when $S_{t}={\left[I_{t|t-1}\right]}^{-1}$, the GAS (1, 1) model results in the standard GARCH model.

#### Incorporating long memory with the GAS model:

Adopting the methods of Janus et al. [20] and Gao and Shi [18], integrating long memory into the original GAS process can be achieved by adjusting $f_{t}$ from the general GAS model of Equation 6 as follows,

(9) $$(1-\beta L)\left(h_{t}-\omega^{*}\right)=\zeta_{t}^{*}$$

where L is the lag operator, β is a scalar persistence parameter (conceptually related to the $\beta_{j}$ coefficients in the GAS recursion) $\omega^{*}=\frac{\omega }{\left(1-\beta \right)}$ measures the unconditional mean of $f_{t}$ and $\zeta_{t}^{*}=\alpha \zeta_{t-1}$. Once the fractional differencing factor is included, we can rewrite this as,

(10) $$(1-\beta L){\left(1-L\right)}^{d}\left(h_{t}-\omega^{*}\right)=\zeta_{t}^{*}.$$

Assuming that |β|<1, the following implications exists for various values of the long memory parameter,d:

- For d<0, all autocovariances (excluding lag 0) are negative indicating anti-persistence, i.e., decaying in a hyperbolic manner to zero.
- For d=0, the ACF exponentially decays implying a demonstration of short memory.
- For 0<d<1/2, the ACF decays at a slow hyperbolic rate and the process exhibits long memory.
- For 1/2≤d<1, the process is mean reverting, however it is not covariance stationary.
- For d=1, $f_{t}$ will follow a unit root process.

The methods of the GAS model used in this analysis were applied utilizing the **GAS** [27] package of the statistical software, **R**.

## 2.3 Heavy-tailed distributions

Heavy-tailed distributions are commonly used for financial modeling as it is able to represent empirical traits such as kurtosis, skewness, extreme events, volatility clustering, etc. which the Normal distribution is unable to capture. The generalized hyperbolic and generalized lambda distributions are derived from different classes of distributions but possess flexible frameworks that are able to sufficiently capture the distinct characteristics of cryptocurrencies.

### 2.3.1 Generalized hyperbolic distributions

The family of generalized hyperbolic distributions (GHD), introduced by Barndorff-Nielsen [28], encompasses various distributions which are highly essential when it comes to modeling financial data. The GHD is achievable through a modification of the generalized inverse Gaussian (GIG) distribution. The random variable X follows a generalized inverse Gaussian (GIG) distribution if its probability density function is given as,

(11) $$h(x;\lambda ,\chi ,\psi )=\frac{\chi^{-\lambda }{\left(\sqrt{\chi \psi }\right)}^{\lambda }}{2K_{\lambda }(\sqrt{\chi \psi })}x^{\lambda -1}\exp\left(-\frac{1}{2}\left(\chi x^{-1}+\psi x\right)\right)$$

for x>0, χψ>0 and $K_{\lambda }$ is a modified Bessel function of the third kind with index λ.

Thus the distribution is dependent on three real parameters:χ, ψ, λ, two parameter vectors: the location parameter (μ) and the skewness parameter (γ) in ${\mathbb{R}}^{d}$, and d×d positive matrix Σ, i.e.,$X\sim GH_{d}(\lambda ,\chi ,\psi ,\mu ,\gamma ,\Sigma )$. If λ=1 in Equation 10, the multivariate generalized hyperbolic distribution is achieved with univariate margins that are one dimensional hyperbolic distributions. This one dimensional distribution is widely used to model univariate financial data [29].

The methods applied for the GH distributions in the statistical software platform **R** are available within the package **ghyp** [30].

### 2.3.2 Generalized lambda distributions

Introduced by Ramberg and Schmeiser [31], the generalized lambda distribution (GLD) is a four-parameter (location, scale, kurtosis and skewness) modification of Tukey’s lambda distribution [32]. The GLD is recognized for it’s flexibility to model a wide range of data, including financial data, however the complexity of the parameter estimation process proves to be a shortfall of this distribution [33]. The traditional parameterizations of the GLD are known as the RS (Ramberg and Schmeiser) [31] parameterization and the FMKL (Freimer–Mudholkar–Kollia–Lin) [34] parameterization.

The FMKL GLD parameterization is given by,

(12) $$Q(u)=\lambda_{1}+\frac{\frac{u^{\lambda_{3}-1}}{\lambda_{3}}-\frac{{\left(1-u\right)}^{\lambda_{4}-1}}{\lambda_{4}}}{\lambda_{2}},$$

where $\lambda_{1}$ is the location parameter, $\lambda_{2}$ is the scale parameter, and, $\lambda_{3}$ and $\lambda_{4}$ are defined as the shape parameters, i.e., $\lambda_{3}$ is a skewness parameter and $\lambda_{4}$ is a kurtosis parameter.

The methods applied for the GLD models in the statistical software platform **R** are available within the package **GLDEX** [35].

## 2.4 VaR and backtesting

In finance, estimating risk measures is crucial as these measures are paramount for traders to assess the risks that are tied with their portfolios’ future values. This permits for the consideration of potential losses. Value-at-Risk (VaR) is the most commonly used risk metric in market risk management. It is assessed at long and short positions. Generally, traders who are selling, i.e., traders at a short position, will encounter a loss if the price increases and traders who are buying, i.e., traders who are at a long position, will face a loss if the price drops. VaR is a summary of the statistical measures of potential losses and is expressed as a confidence interval in units of a specific currency over a specific time period. If $\hat{O}(\cdot )$ represents the cumulative distribution function (cdf) of the most appropriate distribution then VaR can be defined as

(13) $$\mathrm{VaR}(p)={\hat{O}}^{-1}(p),$$

for 0<p<1.

Backtesting the adequacy of a model involves the recursive approach of forecasting [36]. This method is also used to compare models in terms of VaR predictions. The aim of backtesting analysis is to evaluate the precision of the forecast by splitting the estimation and evaluation period. VaR backtesting processes evaluate the correct coverage of the unconditional and conditional left-tail of a log returns distribution [37]. The correct unconditional coverage (UC) was first considered by Kupiec [38] and the correct conditional coverage (CC) was first considered by Christoffersen [39].

In the section to follow, the long memory models (FIAPARCH and LMGAS models) are fitted to the returns of all four cryptocurrencies. Once this is achieved, the standardized residuals of the fitted models are extracted. The heavy-tailed distributions discussed above are then fitted to the residuals to allow for an enhanced representation of the underlying dynamics in these cryptocurrency markets. VaR is subsequently investigated and backtesting is conducted to assess the robustness of the fitted models in evaluating risks and performing predictions.

## 3 Empirical results

This section describes the results that are produced by applying the long memory volatility models discussed in the previous section to the returns of Bitcoin, Ethereum, Litecoin, and Ripple. The analysis highlights each model’s performance in terms of their ability to represent the cryptocurrencies and the models’ robustness in assessing risk and making predictions through fitting heavy-tailed distributions to capture underlying dynamics.

### 3.1 Data source and description

The daily Bitcoin, Ethereum, Litecoin, and Ripple closing prices (based on the volume-weighted average price, VWAP) in United States dollars (USD) are the datasets used in this analysis. All data sets were obtained from https://www.coingecko.com/en/. Two thousand five hundred and fifty six daily observations were used for the Bitcoin, Litecoin, and Ripple closing prices for the period 07/08/2015–31/07/2022 and 2,550 daily observations were used for the Ethereum closing prices. The time periods for Ethereum differ from the other cryptocurrencies used in this study due to availability constraints. This does not impact our results as we focus on the capabilities of each model to capture the underlying trends exhibited by each cryptocurrency.

**FIGURE 1:**
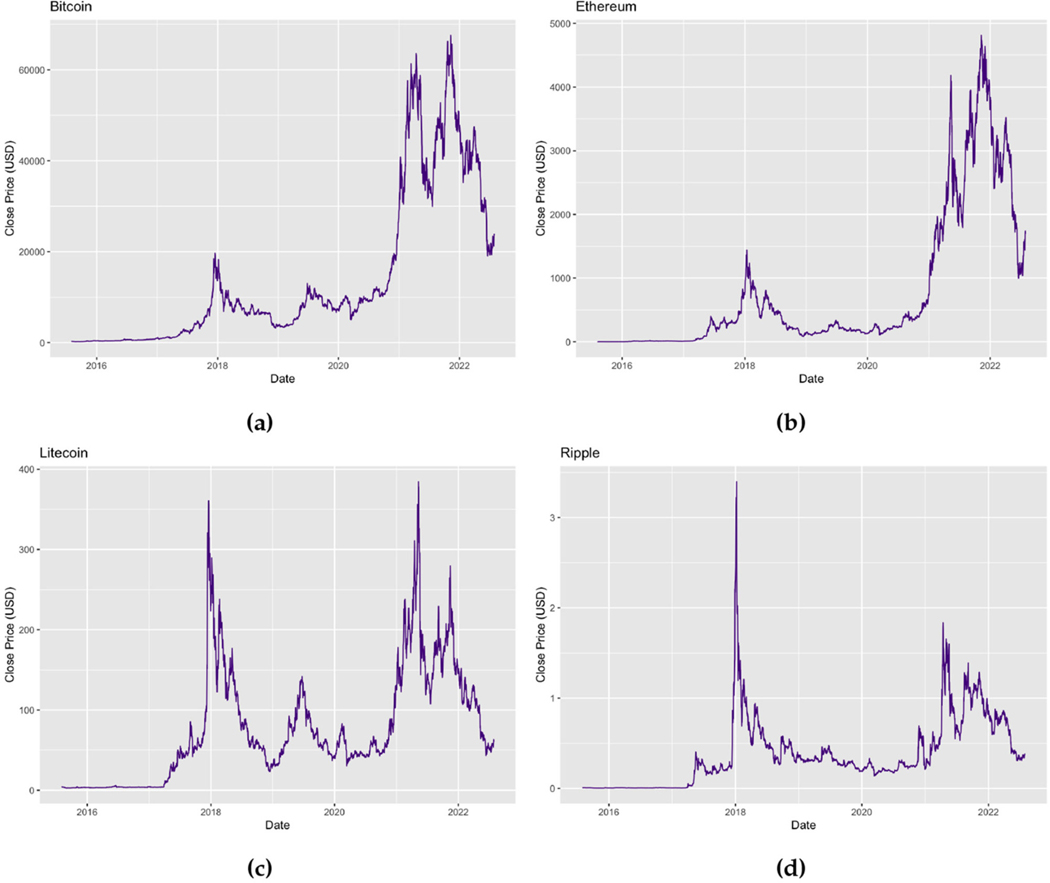
Time series plots of (a) daily Bitcoin prices (USD) for the period 01/08/2015–31/07/2022, (b) daily Ethereum prices (USD) for the period 07/08/2015–31/07/2022, (c) daily Litecoin prices (USD) for the period 01/08/2015–31/07/2022, and (d) daily Ripple prices (USD) for the period 01/08/2015–31/07/2022.

Figure 1 depicts the general trends of the time series plots of the closing prices (USD) for Bitcoin, Ethereum, Litecoin, and Ripple. By inspecting the plots, it can be deduced that the daily prices of the cryptocurrencies may exhibit non-constant means as well as high variability for the considered periods. This is consistent to what is expected of financial data, however, investors of cryptocurrencies are ideally interested in the returns of their investments and thus, the daily prices are converted to log returns, $r_{t}$, as follows,

(14) $$r_{t}=\ln\frac{p_{t}}{p_{t-1}}$$

where $p_{t}$ is the closing price of the cryptocurrency at time t, and $p_{t-1}$ is the closing price of the cryptocurrency at time t−1.

**FIGURE 2:**
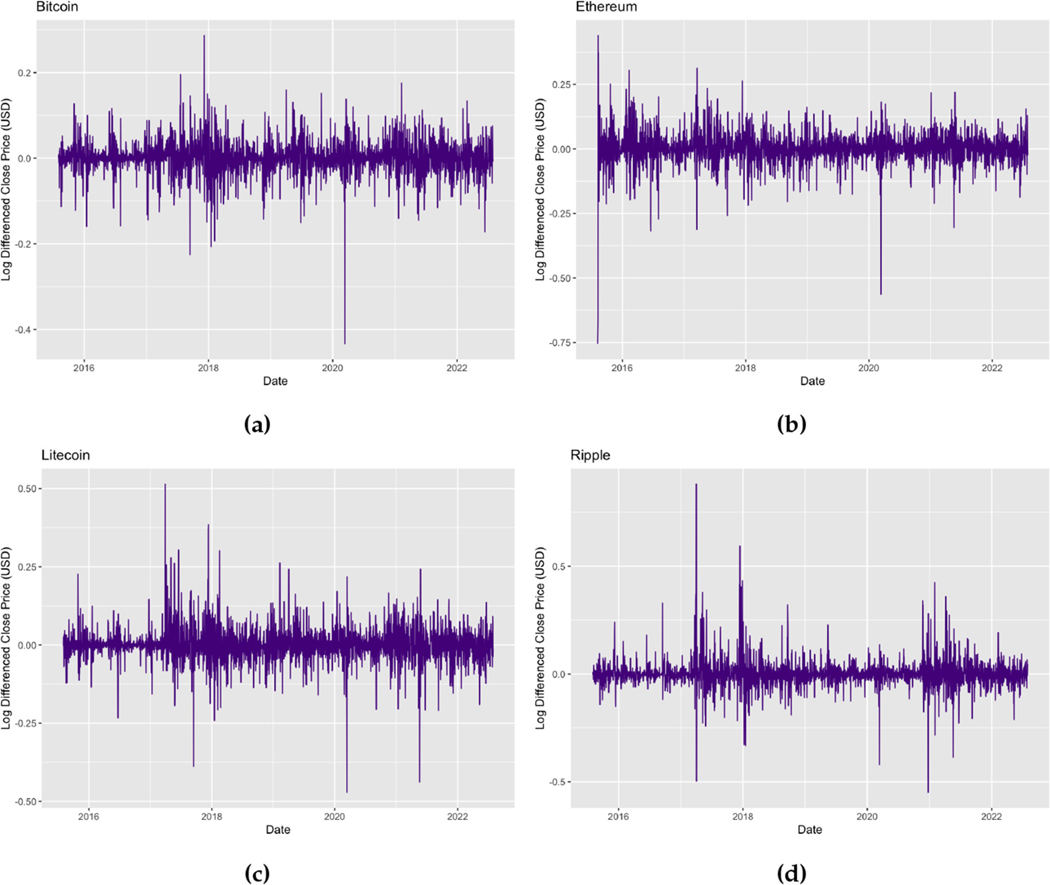
Time series plots of (a) daily Bitcoin log returns for the period 01/08/2015–31/07/2022, (b) daily Ethereum log returns for the period 07/08/2015–31/07/2022, (c) daily Litecoin log returns for the period 01/08/2015–31/07/2022, and (d) daily Ripple log returns for the period 01/08/2015–31/07/2022.

Figure 2 displays the time series plots of the log returns of the daily Bitcoin, Ethereum, Litecoin, and Ripple prices. The plots show that the log returns of all four cryptocurrencies fluctuate around zero implying that the means of the returns are now stationary, however a time-varying variance may still be persistent indicating volatility clustering.

**FIGURE 3:**
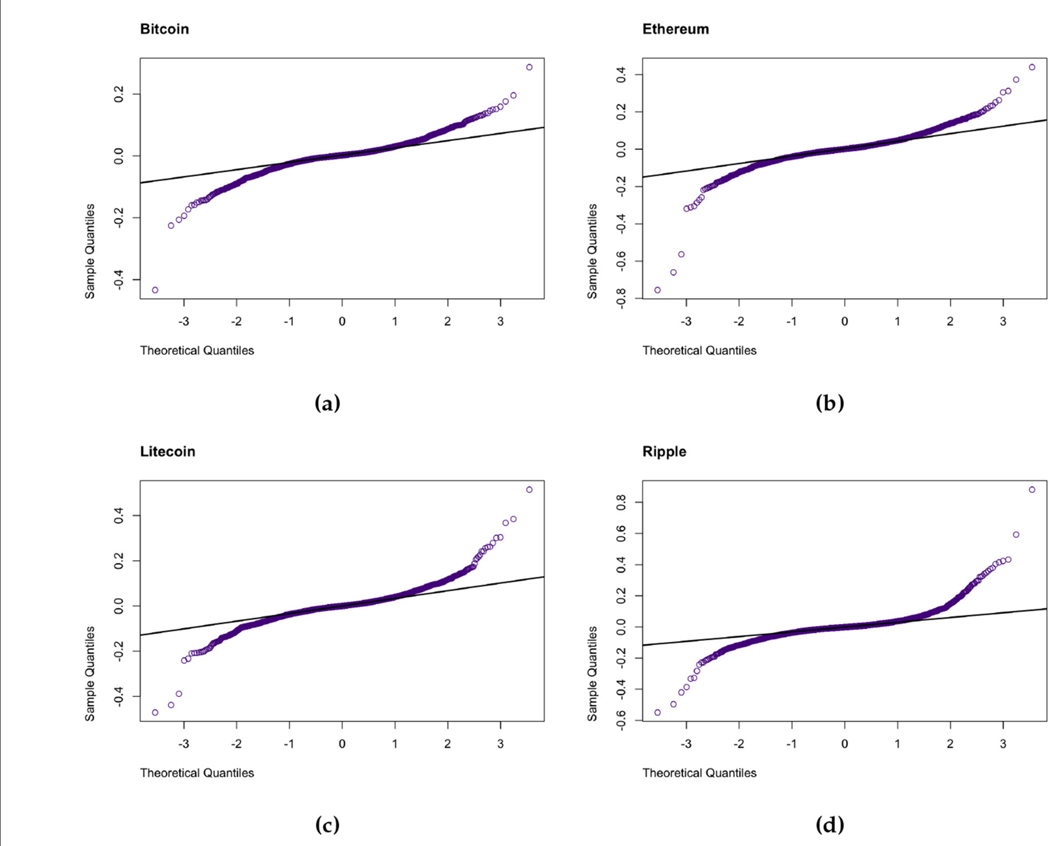
QQ plots of the daily (a) Bitcoin, (b) Ethereum, (c) Litecoin, and (d) Ripple.

Descriptive statistics and the results of the formal tests applied to the cryptocurrencies are presented in Table 1. The mean returns across all assets are slightly positive, indicating slight upward trends throughout the period. However, these trends are relatively small in magnitude, which is typical behavior for daily financial returns. Asymmetry in the return distributions is revealed by the skewness statistics. Bitcoin and Ethereum exhibit negative skewness which suggests that the left tails are heavier than the right tails i.e., a higher probability of large negative returns (left tail risk), while Litecoin and Ripple are both positively skewed and thus indicating more frequent large positive returns. This indicates contrasting investor behaviors and possibly different degrees of speculative trading between the cryptocurrencies. For instance, Bitcoin and Ethereum have been around longer and are more widely held and thus, may exhibit sharper downside corrections during market stress when compared to Litecoin and Ripple. All cryptocurrencies’ returns are strongly leptokurtic as indicated by the positive excess kurtosis. This is also verified by the QQ-plots in Figure 3 as well as the Jarque-Bera [40] and Shapiro Wilk [41] tests of normality which suggest the data are not normally distributed. The fat tails exhibited by the four sets of returns confirm the presence of extreme events and support the choice of heavy-tailed distributions like the GHD and GLD in our chosen modeling framework. The presence of tail risk is especially relevant in cryptocurrency markets due to their vulnerability to sharp crashes, exchange collapses, as well as sudden regulatory announcements. The stationarity of the returns was tested by the three cases of the Augmented Dickey-Fuller (ADF) [42], Phillips-Perron (PP) [43], and Kwiatkowski-Phillips-Schmidt-Shin (KPSS tests) [44]. These tests mostly support the assumption that the returns are stationary at the 5% level of significance; however for Bitcoin and Ethereum under KPSS, no trend, the case indicates that these assets may contain weak non-stationary components which could possibly be driven by structural changes over time. The Ljung-Box [45] test performed on the returns suggests a lack of autocorrelation, while the test performed on the squared returns reveals significant serial correlation. This is a defining trait of volatility clustering. The ARCH-LM confirms the presence of conditional heteroskedasticity which validates our approach to employ GARCH-type volatility models. Lastly, time variation of the returns is investigated using the Cox-Stuart [46] test, which fails to detect montonic time trends, validating the assumption of stationarity in means. The exploratory data analysis confirms that the Bitcoin, Ethereum, Litecoin and Ripple returns exhibit stylized facts commonly observed in financial data, like volatility clustering, heavy tails and non-linear dependence, as outlined by Cont [47].

Long memory tests were performed to investigate the long memory properties that may be exhibited by the returns and their squared counterparts. The Rescaled Range (R/S) statistic proposed by Hurst [48] and the Geweke and Porter-Hudak (GPH) model [49] were used for the LM testing approach in this analysis. The results of the LM tests performed is found in Table 2. The Hurst exponent of the R/S analysis, *H*, denotes the measure of the long memory behavior demonstrated by the time series. The resulting *H* values for the returns of all four cryptocurrencies are >0.6 implying mild persistence, while values above 0.65 in the squared returns indicate much stronger long memory in volatility. This indicates that volatility shocks in the cryptocurrency markets gradually decay over time. This is a characteristic consistent with prolonged periods of high or low volatility. The GPH estimator offers supplementary results by estimating the fractional differencing parameter (*d*), with values between 0 and 0.5 confirming long memory. Consistent with the R/S test, the *d* estimates are higher for the squared returns, reinforcing that the volatility processes of these assets exhibit stronger persistence than their returns. It appears that the relatively high long memory values in squared returns, especially for Litecoin and Ripple, may reflect the influence of speculative trading, low liquidity, or fragmented market structure, which can exacerbate volatility persistence in smaller coins in the market.

Thus, the results of these tests substantiate the notion that standard volatility models, which fail to cater for the long memory component, may not be adequate for cryptocurrencies as these models could underestimate the persistence of risk over time. Comprehending how these dynamics work is critical for investors and risk managers, as it implies that risk does not revert promptly after a shock. This reinforces the case for adopting long-memory volatility models, which can better account for the slowly decaying serial correlations observed in these markets.

### 3.2 Model specification and estimation

The long memory GAS model and the FIAPARCH model with normal innovations are fitted to the four sets of returns. For a comparative benchmark, a standard GAS and GARCH model with normal innovations are also estimated. The GAS and LMGAS model are fitted to the returns with various specifications, based on the Akaike Information Criterion (AIC), of the parameters for location (μ), scale (ϕ), skewness (γ), and shape (v) (1 and/or 2) to achieve adequate fits. The parameter estimates (reported in Appendix A) are statistically significant for all models across the four cryptocurrencies, with long memory models (particularly FIAPARCH and LMGAS) exhibiting higher persistence in volatility. These models also tend to assign more weight to past observations, aligning with the established presence of long memory in these assets.

The standardized residuals are extracted from the GAS, LMGAS, GARCH, and FIAPARCH models for further analysis. Assessing the residuals is an essential step in ensuring that the fitted models are adequate. Typically, the residuals of financial return models should behave similar to that of white noise. However, if the residuals are found to violate normality assumptions, heavy-tailed distributions may be required to capture any underlying dynamics of the innovations. The residuals from all four models display features inconsistent with normality, such as excess kurtosis and skewness which is confirmed by Jarque-Bera test results, which reject the null hypothesis of normality for all assets and models (see Appendix B). These results suggest that Gaussian innovations may not be adequate to capture the heavy-tailed behavior of cryptocurrency returns. This warrants the use of the heavy-tailed innovation distributions, the Generalized Hyperbolic Distribution (GHD) and the Generalized Lambda Distribution (GLD). The adequacy of these fits were evaluated using the Anderson-Darling (AD) [50] test (Appendix C). The results imply that the GHD and GLD provide significantly better fits to the residual distributions in the GAS, LMGAS, and FIAPARCH models, however the GARCH model remains inadequate. This could be due to the GARCH model’s limited robustness in capturing complex dynamics.

Overall, this two-step approach of fitting the models under Gaussian assumptions and thereafter refining them to adopt heavy-tailed innovations ensures that the chosen models are both conceptually valid and empirically well-suited to the unique features of cryptocurrency volatility.

### 3.3 Value-at-Risk estimation

Value-at-Risk (VaR) is a commonly used risk measure that provides an estimate of the potential losses over a period of time at a given confidence interval. In this study, we produce VaR estimates at commonly used quantile levels 1%, 2.5%, and 5% for the long positions (left tail risk), and 95%, 97.5%, and 99% for short positions (right tail risk). A detailed breakdown of VaR estimates for the fitted models is reported in Appendix C. Table 3 below summarizes the models that produced the lowest VaR estimates, i.e., most conservative forecast, at the long and short positions across the different cryptocurrencies.

From a financial perspective, these results provide valuable insights into how the different volatility models behave across different risk quantiles and asset types. For majority of the short position quantiles where large upward price movements can pose risk to short sellers, the LMGAS models tend to produce the lowest VaR estimates. This suggests a stronger sensitivity to extreme right-tail risks and thus indicates that the long memory component is especially effective in capturing the volatility persistence that often occur in speculative runs or short squeezes which are commonly observed in cryptocurrency markets. Notably, the LMGAS-GHD model dominates the short-side risk estimates for Bitcoin and Ripple at extreme quantiles. This reflects its ability to detect and prepare for high-magnitude positive shocks. The LMGAS-GLD model appears most conservative for Ethereum and Litecoin which could be indicative of unique trading patterns and skewness in returns, especially during periods of protocol upgrades or retaildriven rallies. For the long positions, the standard GAS and GARCH models appear to result in low VaR values which may indicate that although these models are less complex, they still have the ability to capture risk under certain distributional conditions, implying that in some stable market regimes these simpler models may suffice for capturing downside risk. However, these models may underestimate risk in turbulent periods.

Thus, these results indicate that model performance varies across cryptocurrencies and positions with long memory structures being particularly valuable in modeling right-tail risk exposures, while performance for downside risk is more mixed. In the following sections, we perform backtesting methods to determine whether these VaR estimates adequately represent actual risk exposure.

### 3.4 In-sample backtesting

The adequacy of the fits of the models was evaluated by in-sample backtesting for the periods 01/08/2015–31/07/2022 for Bitcoin, Litecoin, and Ripple and 07/08/2015–31/07/2022 for Ethereum, using two formal statistical methods, the Kupiec likelihood ratio test, which examines the unconditional coverage of VaR exceedances, and the Christoffersen conditional coverage test, which furthers the analysis to add the independence of exceedance sequences. Table 4 summarizes the best performing models for each cryptocurrency for multiple risk levels, based on the highest *p*-values from both the Kupiec and Christoffersen tests. A model is considered to perform best for a given cryptocurrency if it consistently produces the highest *p*-values across both tests, particularly at the extreme tails (1% and 99%), where risk assessment is most critical. A higher *p*-value, exceeding 0.05 suggests that the model’s VaR forecasts are statistically consistent with the observed violations and thus is more more reliable for risk management.

The results of backtesting offer key information on the practical relevance of each model in managing cryptocurrency risk. Although the long memory GAS models perform adequately at certain risk levels, they appear to fail to capture extreme tail risk which is primarily what risk managers and regulators are most concerned with. This is reflected in the low *p*-values, especially at the 1% and 99% quantiles. This implies that the model’s capabilities for VaR prediction may be limited in that even though they are able to capture some aspects of volatility persistence, they tend to struggle with abrupt shocks exhibited by cryptocurrencies unless paired with distributions that can better capture tail behavior. Conversely, the FIAPARCH-GLD and GARCH-type models demonstrate more stable performance across the risk levels. These models appear to be better equipped to handle the significant kurtosis and volatility exhibited by cryptocurrencies. Particularly, for Bitcoin and Litecoin, the FIAPARCH-GLD model provides the most reliable in-sample results, indicating its robustness in modeling downside risk and volatility clustering which are features often observed during market stress or speculative bubbles in these assets. The flexibility demonstrated by the FIAPARCH-GLD model in including both long memory and asymmetric responses to shocks makes it particularly appropriate for markets like Bitcoin where price swings can be sudden and prolonged. For Ethereum, the GARCH-GLD model outperforms others, specifically at higher quantiles, indicating that despite its strong technical fundamentals and investor diversity, a relatively simpler volatility model paired with a heavy-tailed distribution is enough to capture its risk structure. For Ripple, both GARCH variants deliver strong and consistent results suggesting that its behavior is not well captured by models with higher parameter complexity.

In-sample findings are essential for portfolio managers and institutional traders who rely on accurate VaR predictions to make capital allocation, set margins, and hedging decisions. The models that perform well under backtesting not only offer statistical adequacy but also align more closely with the specific risk profiles and trading behavior of each asset. For further evaluation on the predictive performance of the models, the following section presents a static out-of-sample backtesting analysis based on forecasts from August 2022 to December 2024.

### 3.5 Out-of-sample backtesting

In order to assess the predictive performance of the models beyond the estimation sample, an out-of-sample evaluation was performed using a reserved forecasting period from 01 August 2022 to 31 December 2024. Similar to the methods of in-sample backtesting, the Kupiec and Christoffersen tests were applied to evaluate the unconditional and conditional coverage properties of the VaR forecasts. The *p*-values for all models across several risk levels for both long and short positions are recorded in Appendix C. A summary of the best-performing models is provided in Table 5.

The results show that the long memory volatility models retain their predictive advantage out of sample. Specifically, for Bitcoin and Litecoin, the FIAPARCH-GHD model exhibits strong tail performance and robust conditional coverage, which portrays its flexibility in capturing long memory and asymmetric volatility, which are generally exhibited during turbulent periods in markets. For Ethereum, both GAS-GHD and FIAPARCH-GHD models perform well, with the GAS-GHD achieving better results at the more extreme quantiles implying that non-long memory models may perform better under certain distributional assumptions, particularly for more mature coins. Ripple’s results indicate that GARCH-GLD and FIAPARCH-GLD models outperform the other models in terms of forecasting, especially for high confidence levels which supports the notion that heavy-tailed distributions are especially useful for modeling assets that are prone to extreme price swings.

Overall, these findings validates the practical value of these models in real-world forecasting and risk management scenarios.

### 3.6 Volatility forecast evaluation

The models’ capability to produce accurate volatility forecasts beyond the sample used for estimation was assessed by utilizing their one-step-ahead conditional variance predictions using the Root Mean Squared Error (RMSE) and the Quasi-Likelihood (QLIKE) metrics. These measures are widely used in volatility modeling, with QLIKE being resilient to noise and is generally more sensitive to under estimated variance.

The volatility forecasts are evaluated over the out-of-sample period from 01 August 2022 to 31 December 2024. We assess the models’ forecast performance based on the accuracy of their conditional variance predictions for this holdout period. Table 6 provides a summary of the best-performing models based on RMSE and QLIKE measures. The detailed results of the RMSE and QLIKE values for all models and cryptocurrencies are found in Appendix C. Lower RMSE and QLIKE values tend to suggest better forecast performance.

For all four cryptocurrencies, the FIAPARCH-GLD and GARCH-type models produce the lowest RMSE and QLIKE values, and thus suggests the most superior forecast accuracy when compared to the LMGAS and GAS-type models. For Bitcoin and Ethereum, the FIAPARCH-GLD model slightly outperforms the other models, with GARCH-GHD and GARCH-GLD also performing strongly. Although the GAS and LMGAS models are highly flexible, these models generate significantly higher loss values. The results of this section reinforce the VaR backtesting results, suggesting that GARCH-type and FIAPARCH models are more reliable for practical volatility forecasting and risk management in cryptocurrency markets.

## 4 Conclusion

In this study, we have investigated and modeled the long memory features of four highly-traded cryptocurrencies, Bitcoin, Ethereum, Litecoin, and Ripple by employing long memory extensions of the GAS and GARCH model. In line with prior research [7, 21], our results confirm that the presence of long memory in cryptocurrencies is prevalent. This implies that the standard GARCH and GAS-type models may fall short when it comes to capturing the persistent long-term dependencies found in the volatility dynamics and thus validating the need for long memory integrated models.

We evaluated the performance of LMGAS models and FIAPARCH models and compared them against benchmark models, standard GAS and GARCH models. By extending traditional methods, we adopted heavy-tailed innovation distributions, i.e., GHD and GLD and therefore introduce LMGAS-GHD, LMGAS-GLD, FIAPARCH-GHD, and FIAPARCH-GLD models.

The adequacy of the models were assessed in multiple stages. The AD-tests show that all the models except the GARCH models were able to capture the distributional properties of the standardized residuals. VaR estimation and in-sample backtesting, using the Kupiec and Christoffersen tests, revealed that FIAPARCH-GLD consistently produced the most desirable results, especially at extreme quantiles. The GARCH models also demonstrated stability, however they tend to under represent extreme risk events. In contrast, the LMGAS models underperformed at extreme risk levels. Nevertheless, they were still able to capture some risk dynamics. The GAS models produced mixed results with numerous fluctuations for the cryptocurrencies and thus may be unstable for risk estimation.

We also extended the analysis by conducting out-of-sample backtesting to evaluate the models’ predictive ability under real-world market conditions. The findings were consistent in that the FIAPARCH-GLD and GARCH-type models consistently produced strong results across multiple VaR quantiles. Additionally, a volatility forecasting evaluation using RMSE and QLIKE metrics confirmed the robustness of these models, particularly FIAPARCHGLD, for all four cryptocurrencies. However, the LMGAS models yielded unstable forecasts, with several undefined QLIKE values and large RMSE values, further implying their limitations in practical forecasting.

Overall, the results of this study reveal that long memory and asymmetric volatility models, especially FIAPARCH with heavy-tailed innovations, provide a powerful framework for both risk estimation and volatility forecasting in cryptocurrency markets. From a financial perspective, our results provide new insights into the volatility behavior of cryptocurrencies. The consistent outperformance of long memory models indicates that risk in cryptocurrency markets may not decay as quickly as in traditional assets. It appears that cryptocurrencies exhibits persistence that, if ignored, could lead to the underestimation of extreme losses. The findings in this paper may be of relevance to all those who are involved and interested in the cryptocurrency market as the explorations in this study develop sophisticated modeling techniques to grasp the complexities of cryptocurrencies. For practitioners, including portfolio managers, institutional traders and risk managers, our findings highlight the importance of selecting models that can robustly capture volatility clustering and tail dependence.

Further recommendations of research includes employing various other heavy-tailed distributions to govern the innovations of the LMGAS and FIGARCH models. Other long memory GARCH models, like the HYGARCH and FIGARCH, can be used to model cryptocurrencies and relative comparisons can be made with the models utilized in this paper. Extending the analysis performed in this study to other popular cryptocurrencies such as Monero, Dogecoin, Tether etc. can also be of great value. Finally, extending this framework using rolling window estimation could provide valuable insights into the dynamic evolution of risk and long memory characteristics in response to external shocks and market events.

## Supplementary Material

Data sheet 1

The Supplementary Material for this article can be found online at: https://www.frontiersin.org/articles/10.3389/fams.2025.1567626/full#supplementary-material

## Figures and Tables

**TABLE 1:** Descriptive statistics and formal tests of the log returns of daily Bitcoin prices (BTC/USD), Ethereum prices (ETH/USD), Litecoin prices (LTC/USD), and Ripple prices (XRP/USD).

Distribution metrics	Test	Bitcoin		Ethereum		Litecoin		Ripple	
		Test statistic	*p*-value	Test Statistic	*p*-value	Test statistic	*p*-value	Statistic	p-value
Mean		0.0017	-	0.0025	-	0.0010	-	0.0015	-
Standard deviation		0.0394	-	0.0631	-	0.0555	-	0.0661	-
Skewness		−0.6334	-	−1.2025	-	0.2166	-	1.6734	-
Kurtosis		9.7834	-	18.5819	-	11.4391	-	24.9938	-
Stationarity	ADF test								
	*Case 1:* No drift, no trend	−18.3000	<0.0100	17.4000	<0.0100	−18.0000	<0.0100	−16.7000	<0.0100
	*Case 2:* Drift, no trend	−18.4000	<0.0100	17.6000	<0.0100	−19.9000	<0.0100	−16.7000	<0.0100
	*Case 3:* Drift and trend	−18.4000	<0.0100	17.6000	<0.0100	−18.1000	<0.0100	−16.8000	<0.0100
	PP test								
	*Case 1:* No drift, no trend	−2,750.0000	<0.0100	−2,522.0000	<0.0100	−2,763.0000	<0.0100	−2,881.0000	<0.0100
	*Case 2:* Drift, no trend	−2,739.0000	<0.0100	−2,511.0000	<0.0100	−2,761.0000	<0.0100	−2,879.0000	<0.0100
	*Case 3:* Drift and trend	−2,735.0000	<0.0100	−2,505.0000	<0.0100	−2,758.0000	<0.0100	−2,878.0000	<0.0100
	KPSS test								
	*Case 1:* No drift, no trend	2.6200	0.0132	2.7200	0.0109	0.8270	0.1000	0.7610	0.1000
	*Case 2:* Drift, no trend	0.2940	0.1000	0.3620	0.0935	0.2030	0.1000	0.1550	0.1000
	*Case 3:* Drift and trend	0.1090	0.0100	0.0866	0.1000	0.0798	0.1000	0.0663	0.1000
Normality	Jarque-Bera test	1.0386×10^4^	<0.0001	3.7354×10^4^	<0.0001	1.3983×10^4^	<0.0001	6.7840×10^4^	<0.0001
	Shapiro-Wilk	0.9108	<0.0001	0.8747	<0.0001	0.8877	<0.0001	0.7791	<0.0001
Time variation	Cox-Stuart test	614.0000	0.1705	667.0000	0.0983	649.0000	0.5951	644.0000	0.8012
Autocorrelation	Ljung-Box test	7.5434	0.3746	8.4898	0.2914	65.6450	0.0680	0.0968	0.7558
ARCH effects	Ljung-Box test $\left(r_{t}^{2}\right)$	19.1620	<0.0001	558.0200	<0.0001	43.0870	<0.0001	165.3900	<0.0001
	ARCH-LM test	18.6550	<0.0001	910.5800	<0.0001	42.3910	<0.0001	165.4600	<0.0001

**TABLE 2:** Long memory tests of the returns of daily Bitcoin prices (BTC/USD), Ethereum prices (ETH/USD), Litecoin prices (LTC/USD), and Ripple prices (XRP/USD).

	Test	Statistic	Estimate
**Returns**			
Bitcoin	R/S analysis	Hurst exponent	0.6090
	GPH test	*d* estimate	0.0230
Ethereum	R/S analysis	Hurst exponent	0.6373
	GPH test	*d* estimate	0.0658
Litecoin	R/S analysis	Hurst exponent	0.6088
	GPH test	*d* estimate	0.0460
Ripple	R/S analysis	Hurst exponent	0.6132
	GPH test	*d* estimate	0.1318
**Squared returns**			
Bitcoin	R/S analysis	Hurst exponent	0.6777
	GPH test	*d* estimate	0.2770
Ethereum	R/S analysis	Hurst exponent	0.6615
	GPH test	*d* estimate	0.0780
Litecoin	R/S analysis	Hurst exponent	0.7045
	GPH test	*d* estimate	0.3047
Ripple	R/S analysis	Hurst exponent	0.7202
	GPH test	*d* estimate	0.3043

**TABLE 3:** Summary of best performing models by lowest VaR estimates.

	Best model (long position)	Best model (short position)
Bitcoin	FIAPARCH-GLD	LMGAS-GHD
Ethereum	GARCH-GHD	LMGAS-GLD
Litecoin	GAS-GHD	LMGAS-GLD
Ripple	GARCH-GHD	LMGAS-GHD

**TABLE 4:** Summary of best-performing models for in-sample VaR accuracy based on Kupiec and Christoffersen tests for the periods 01/08/2015–31/07/2022 for Bitcoin, Litecoin, and Ripple and 07/08/2015–31/07/2022 for Ethereum.

	Best model(s)	Observations
Bitcoin	FIAPARCH-GLD	Strong tail performance and good conditional coverage
Ethereum	FIAPARCH-GLD, GARCH-GLD	Consistent across long and short positions
Litecoin	GARCH-GHD, FIAPARCH-GLD	Robust across quantiles
Ripple	GARCH-GHD, GARCH-GLD	Best unconditional and conditional coverage

**TABLE 5:** Summary of best-performing models for out-of-sample VaR accuracy for the period 01/08/2022–31/12/2024 based on Kupiec and Christoffersen tests.

Cryptocurrency	Best model(s)	Observations
Bitcoin	FIAPARCH-GHD	Strong tail accuracy and best performance under the Christoffersen test, especially at the extreme quantiles.
Ethereum	GAS-GHD, FIAPARCH-GHD	GAS-GHD performs better at extreme quantiles whereas FIAPARCH-GHD shows consistent coverage across levels.
Litecoin	FIAPARCH-GHD	Best performance at both tails and strong conditional coverage at most quantiles.
Ripple	GARCH-GLD, FIAPARCH-GLD	Reliable forecasts at high confidence levels with strong *p*-values for both tests.

**TABLE 6:** Best-performing models for out-of-sample volatility forecasting based on RMSE and QLIKE.

Cryptocurrency	Best model(s)	Observations
Bitcoin	FIAPARCH-GLD, GARCH-GLD	Lowest RMSE and QLIKE values
Ethereum	FIAPARCH-GLD, GARCH-GHD	Strong and consistent accuracy
Litecoin	FIAPARCH-GLD, GARCH-GHD	Robust across both metrics
Ripple	FIAPARCH-GLD	Best overall forecast performance

## Data Availability

Publicly available datasets were analyzed in this study. This data can be found here: https://www.coingecko.com/en/.
