# Supplementary material for: Value at Risk long memory volatility models with heavy-tailed distributions for cryptocurrencies
Source: Front Appl Math Stat. Author manuscript; Available in PMC 2026 Feb 27. (PMC12945339; doi:10.3389/fams.2025.1567626)
Supplement: Data sheet 1 [file NIHMS2096459-supplement-Data_sheet_1.pdf]

Appendix A: Parameter Estimates of Fitted Models

Table 1: Parameter estimation of results of the GAS and LMGAS model for the Bitcoin, Ethereum, Litecoin and Ripple daily returns.

	Parameters	Estimate	p-value
**GAS**			
**Bitcoin**	$\hat{\omega}_\mu$	0.0026	< 0.0001
	$\hat{\omega}_\phi$	-0.6472	< 0.0001
	$\hat{a}_\phi$	0.1898	< 0.0001
	$\hat{b}_\phi$	0.9000	< 0.0001
**Ethereum**	$\hat{\omega}_\mu$	0.0008	< 0.0001
	$\hat{\omega\phi}$	-0.5679	< 0.0001
	$\hat{a}_\phi$	0.1898	< 0.0001
	$\hat{b}_\phi$	0.8972	< 0.0001
**Litecoin**	$\hat{\omega}_\mu$	0.0005	< 0.0001
	$\hat{\omega\phi}$	-0.7858	< 0.0001
	$\hat{a}_\phi$	0.1897	< 0.0001
	$\hat{b}_\phi$	0.8641	< 0.0001
**Ripple**	$\hat{\omega}_\mu$	-0.0032	0.0008
	$\hat{\omega}_\phi$	-0.3809	< 0.0001
	$\hat{a}_\phi$	0.1897	< 0.0001
	$\hat{b}_\phi$	0.9303	< 0.0001
**LMGAS**			
**Bitcoin**	$\hat{\omega}_\mu$	-0.0014	< 0.0001
	$\hat{\omega}_\phi$	-3.6090	< 0.0001
	$\hat{a}_\mu$	0.0070	< 0.0001
	$\hat{b}_\mu$	0.9389	< 0.0001
**Ethereum**	$\hat{\omega}_\mu$	0.0179	< 0.0001
	$\hat{\omega}_\phi$	-1.4072	< 0.0001
	$\hat{a}_\phi$	< 0.0001	< 0.0001
	$\hat{b}_\phi$	0.5661	< 0.0001
**Litecoin**	$\hat{\omega}_\mu$	-0.0002	< 0.0001
	$\hat{\omega}_\phi$	-0.3574	< 0.0001
	$\hat{a}_\mu$	0.0001	< 0.0001
	$\hat{a}_\phi$	0.3794	< 0.0001
	$\hat{b}_\mu$	0.9800	< 0.0001
	$\hat{b}_\phi$	0.9138	< 0.0001
**Ripple**	$\hat{\omega}_\mu$	0.0012	< 0.0001
	$\hat{\omega}_\phi$	-0.7383	< 0.0001
	$\hat{a}_\mu$	< 0.0001	< 0.0001
	$\hat{a}_\phi$	< 0.0001	< 0.0001
	$\hat{b}_\mu$	0.9000	< 0.0001
	$\hat{b}_\phi$	0.7979	< 0.0001

Table 2: Parameter Estimation of results of the GARCH and FIAPARCH model for the Bitcoin, Ethereum, Litecoin and Ripple daily returns.

	Parameters	Estimate	p-value
**GARCH**			
**Bitcoin**	μ	0.0022	0.0008
	ω	0.0001	< 0.0001
	$ARCH(\alpha_1)$	0.1394	< 0.0001
	$GARCH(\beta_1)$	0.8278	< 0.0001
**Ethereum**	μ	0.0019	0.0552
	ω	0.0002	< 0.0001
	$ARCH(\alpha_1)$	0.1642	< 0.0001
	$GARCH(\beta_1)$	0.7594	< 0.0001
**Litecoin**	ω	0.0001	< 0.0001
	$ARCH(\alpha_1)$	0.0740	< 0.0001
	$GARCH(\beta_1)$	0.9027	< 0.0001
**Ripple**	μ	-0.0022	0.0102
	ω	0.0003	< 0.0001
	$ARCH(\alpha_1)$	0.3392	< 0.0001
	$GARCH(\beta_1)$	0.6456	< 0.0001
**FIAPARCH**			
**Bitcoin**	μ	0.0022	0.0010
	$d - FIGARCH(d)$	0.3598	0.0005
	$ARCH(\alpha_1)$	0.2066	0.0681
	$APARCH(\delta_1)$	2.3197	< 0.0001
**Ethereum**	μ	0.0023	0.0467
	$d - FIGARCH(d)$	0.2974	0.0245
	$APARCH(\delta_1)$	2.5429	< 0.0001
**Litecoin**	$d - FIGARCH(d)$	0.3823	0.0757
	$ARCH(\alpha_1)$	0.3354	0.0160
	$GARCH(\beta_1)$	0.6462	0.0112
	$APARCH(\delta_1)$	2.3055	< 0.0001
**Ripple**	μ	-0.0024	0.0437
	$d - FIGARCH(d)$	0.3190	0.0609
	$APARCH(\delta_1)$	2.4313	< 0.0001

Appendix B: Additional Diagnostics

Table 3: Descriptive statistics and normality tests of the residuals extracted from the Gaussian GAS, LMGAS, GARCH and FIAPARCH models fitted to the daily Bitcoin, Ethereum, Litecoin and Ripple returns

	**Bitcoin**		**Ethereum**		**Litecoin**		**Ripple**	
**Statistic/Test**	**Estimate**	**p-value**	**Estimate**	**p-value**	**Estimate**	**p-value**	**Estimate**	**p-value**
**GAS**								
Mean	-0.0267	-	0.0168	-	0.0020	-	0.0622	-
Standard Deviation	0.9712	-	0.9474	-	0.9697	-	0.9207	-
Skewness	-0.6673	-	-1.1450	-	0.5241	-	0.9671	-
Kurtosis	8.6313	-	13.7946	-	14.8150	-	13.8563	-
Jarque-Bera test	8140.9000	< 0.0001	20807.0000	< 0.0001	23536.0000	< 0.0001	20886.0000	< 0.0001
**LMGAS**								
Mean	0.0444	-	0.0155	-	0.0191	-	0.0000	-
Standard Deviation	0.4475	-	0.9994	-	0.9943	-	1.0001	-
Skewness	-26.0515	-	-6.8075	-	-11.1478	-	-2.1435	-
Kurtosis	1036.3681	-	106.8667	-	351.5714	-	56.0263	-
Jarque-Bera	1.1490×10^8	< 0.0001	1.2351×10^6	< 0.0001	1.3242×10^7	< 0.0001	2.5926×10^5	< 0.0001
**GARCH**								
Mean	-0.0132	-	-0.0040	-	0.0165	-	0.0438	-
Standard Deviation	0.9998	-	1.0280	-	0.9993	-	0.9985	-
Skewness	-0.6953	-	-0.9993	-	0.8610	-	0.7898	-
Kurtosis	9.9680	-	11.2900	-	17.8300	-	11.6500	-
Jarque-Bera	10810.000	< 0.0001	13991.0000	< 0.0001	34248.0000	< 0.0001	14744.0000	< 0.0001
**FIAPARCH**								
Mean	-0.0114	-	-0.0144	-	0.0186	-	0.0542	-
Standard Deviation	1.0137	-	1.0003	-	1.0158	-	0.9933	-
Skewness	-0.7083	-	-0.9683	-	1.6594	-	1.3780	-
Kurtosis	10.9311	-	9.8733	-	33.2556	-	21.4419	-
Jarque-Bera	1.2965×10^4	< 0.0001	1.0773×10^4	< 0.0001	1.1916×10^5	< 0.0001	4.9861×10^4	< 0.0001

Table 4: AD tests of the models fitted to the extracted residuals of the Gaussian GAS model from the daily Bitcoin, Ethereum, Litecoin and Ripple log returns

	**Bitcoin**		**Ethereum**		**Litecoin**		**Ripple**	
	**Test Statistic**	**p-value**	**Test Statistic**	**p-value**	**Test Statistic**	**p-value**	**Test Statistic**	**p-value**
**GAS**								
**GHD**	0.5532	0.6932	0.8836	0.4245	0.1999	0.9905	1.6584	0.1428
**GLD**	1.7000	0.1000	1.5000	0.2000	0.6400	0.6000	0.2900	0.9000
**LMGAS**								
**GHD**	1.8000	0.1000	3.2000	0.2000	1.7000	0.1000	1.8000	0.1000
**GLD**	1.4000	0.2000	3.7000	0.1000	1.2000	0.3000	1.8000	0.1000
**GARCH**								
**GHD**	2644	< 0.0001	2576	< 0.0001	2556	< 0.0001	2560	< 0.0001
**GLD**	2653	< 0.0001	2582	< 0.0001	2586	< 0.0001	2561	< 0.0001
**FIAPARCH**								
**GHD**	0.4500	0.8000	0.9400	0.4000	0.2300	1.0000	0.2500	1.0000
**GLD**	1.2000	0.3000	1.4000	0.2000	0.2300	1.0000	0.2600	1.0000

Appendix C: Comprehensive Value-at-Risk (VaR) and Backtesting Tables

Table 5: VaR estimates for the returns at long and short positions

	Distribution	Long position			Short position		
		1%	2.5%	5%	95%	97.5%	99%
**Bitcoin**	GAS-GHD	-2.8720	-2.1247	-1.5803	1.4797	1.9953	2.7030
	GAS-GLD	-3.0842	-2.0929	-1.4982	1.3858	1.9033	2.7357
	LMGAS-GHD	-0.7368	-0.4899	-0.3438	0.4325	0.5690	0.7932
	LMGAS-GLD	-0.7228	-0.4910	-0.3458	0.4425	0.5861	0.8148
	GARCH-GHD	-2.9361	-2.1575	-1.5963	1.5194	2.0472	2.7794
	GARCH-GLD	-3.0905	-2.1132	-1.5193	1.4360	1.9561	2.7833
	FIAPARCH-GHD	-2.9646	-2.1737	-1.6057	1.5416	2.0820	2.8342
	FIAPARCH-GLD	-3.1047	-2.1243	-1.5283	1.4579	1.9921	2.8466
**Ethereum**	GAS-GHD	-2.5471	-1.9138	-1.4484	1.5239	2.0156	2.6848
	GAS-GLD	-2.6234	-1.8913	-1.4114	1.4375	1.9151	2.6428
	LMGAS-GHD	-1.9413	-1.5124	-1.1919	1.4106	1.7267	2.1485
	LMGAS-GLD	-2.9668	-1.9177	-1.3071	1.0565	1.3022	1.6364
	GARCH-GHD	-2.8189	-2.1006	-1.5874	1.5980	2.1260	2.8658
	GARCH-GLD	-2.8649	-2.0829	-1.5659	1.5412	2.0469	2.8075
	FIAPARCH-GHD	-2.7890	-2.0778	-1.5673	1.5500	2.0690	2.7923
	FIAPARCH-GLD	-2.8497	-2.0545	-1.5359	1.4800	1.9738	2.7214
**Litecoin**	GAS-GHD	-2.7293	-1.9667	-1.4390	1.4796	2.0370	2.8438
	GAS-GLD	-2.7507	-1.9061	-1.3854	1.4113	1.9637	2.8734
	LMGAS-GHD	-2.5276	-1.8527	-1.4021	1.5220	2.1192	3.0506
	LMGAS-GLD	-2.1747	-1.6405	-1.2558	1.4175	1.7618	2.2263
	GARCH-GHD	-2.7817	-1.9517	-1.4214	1.5319	2.1300	3.0846
	GARCH-GLD	-2.7458	-1.9312	-1.4182	1.4595	2.0115	2.9046
	FIAPARCH-GHD	-2.7327	-1.9075	-1.3903	1.4663	2.0338	2.9578
	FIAPARCH-GLD	-2.7134	-1.9044	-1.3955	1.4570	2.0060	2.8949
**Ripple**	GAS-GHD	-2.5263	-1.6982	-1.1907	1.4535	2.0911	3.1771
	GAS-GLD	-2.4296	-1.6486	-1.1761	1.3744	1.9549	2.9660
	LMGAS-GHD	-2.6560	-1.8135	-1.3052	1.3586	1.9414	2.9426
	LMGAS-GLD	-2.6019	-1.7939	-1.3007	1.3734	1.9534	2.9435
	GARCH-GHD	-2.7145	-1.8843	-1.3500	1.5275	2.1633	3.1705
	GARCH-GLD	-2.6850	-1.8400	-1.3246	1.4893	2.1142	3.1901
	FIAPARCH-GHD	-2.5547	-1.7818	-1.2905	1.5040	2.1273	3.1473
	FIAPARCH-GLD	-2.5083	-1.7532	-1.2797	1.4836	2.0901	3.1205

Table 6: In-sample VaR backtesting for the returns

	Distribution	Unconditional Coverage						Conditional Coverage					
		Long position			Short position			Long position			Short position		
		1%	2.5%	5%	95%	97.5%	99%	1%	2.5%	5%	95%	97.5%	99%
**Bitcoin**	GAS-GHD	0.2180	0.8089	0.9855	0.1192	0.3733	0.2505	0.312	0.8785	0.5943	0.2565	0.5581	0.1813
	GAS-GLD	0.7540	0.8895	0.4612	0.4090	0.4478	0.2505	0.7583	0.8461	0.2932	0.1738	0.6381	0.1813
	LMGAS-GHD	0.4673	0.8920	0.3655	0.0742	0.5256	0.0228	0.0001	0.0049	0.0085	0.0000	0.0087	0.0155
	LMGAS-GLD	0.6033	0.8920	0.2750	0.2772	0.7089	0.0228	0.0000	0.0000	0.0000	0.0000	0.0125	0.0155
	GARCH-GHD	0.5032	0.8895	0.5337	0.2377	0.5295	0.6047	0.5729	0.1815	0.7122	0.4649	0.7153	0.3891
	GARCH-GLD	0.7540	0.6968	0.5171	0.4612	0.6177	0.6047	0.7583	0.7572	0.2926	0.4481	0.4296	0.3891
	FIAPARCH-GHD	0.6329	0.6177	0.4747	0.3204	0.3067	0.7540	0.6542	0.2085	0.6535	0.3252	0.2255	0.4539
	FIAPARCH-GLD	0.9110	0.9090	0.4612	0.8422	0.7113	0.6047	0.7762	0.202	0.73	0.4216	0.4801	0.3891
**Ethereum**	GAS-GHD	0.4942	0.8260	0.8233	0.9674	0.1613	0.1761	0.5735	0.8892	0.4928	0.0464	0.2751	0.3472
	GAS-GLD	0.9220	0.8260	0.2612	0.0940	0.4609	0.0237	0.6565	0.8892	0.2722	0.0187	0.0749	0.3472
	LMGAS-GHD	0.2897	0.0265	0.3027	0.9638	0.6826	0.6129	0.0000	0.0000	0.0000	0.0000	0.0000	0.0000
	LMGAS-GLD	0.2897	0.0473	0.8918	0.9638	0.3051	0.9210	0.0000	0.0000	0.0000	0.0000	0.0000	0.0000
	GARCH-GHD	0.3825	0.6332	0.8173	0.8173	0.1246	0.2561	0.4834	0.7879	0.6225	0.0279	0.0877	0.4479
	GARCH-GLD	0.9194	0.8260	0.4966	0.3006	0.7277	0.4768	0.7689	0.5176	0.6098	0.0483	0.4861	0.641
	FIAPARCH-GHD	0.9194	0.5439	0.9674	0.6809	0.2572	0.0237	0.7689	0.2104	0.9922	0.8276	0.1564	0.0709
	FIAPARCH-GLD	0.9220	0.8260	0.6164	0.0683	0.9266	0.1155	0.7846	0.2132	0.8812	0.1878	0.8835	0.2549
**Litecoin**	GAS-GHD	0.9110	0.3733	0.8422	0.4193	0.4478	0.1125	0.7762	0.6512	0.2488	0.4234	0.113	0.2498
	GAS-GLD	0.7540	0.4478	0.2051	0.5171	0.7913	0.1125	0.7583	0.7179	0.0493	0.2926	0.1009	0.2498
	LMGAS-GHD	0.0697	0.0031	0.0003	0.2359	0.0246	0.2497	0.1197	0.1751	0.3473	0.0008	0.0018	0.0005
	LMGAS-GLD	0.0229	0.0001	< 0.0001	0.2008	0.0114	0.0411	0.0002	0.0000	0.0000	0.0003	0.0001	0.0008
	GARCH-GHD	0.9110	0.7913	0.5765	0.1433	0.2482	0.1719	0.7762	0.8718	0.0689	0.4733	0.2121	0.6352
	GARCH-GLD	0.9110	0.6071	0.5765	0.9134	0.6968	0.6047	0.7762	0.6985	0.0843	0.1215	0.3004	0.709
	FIAPARCH-GHD	0.9110	0.4465	0.3159	0.8699	0.8089	0.3496	0.7762	0.2972	0.096	0.1811	0.2121	0.5425
	FIAPARCH-GLD	0.9110	0.4465	0.3159	0.9421	0.7912	0.7540	0.1126	0.0017	0.0000	0.0000	0.0003	0.0012
**Ripple**	GAS-GHD	0.4685	0.5295	0.6391	0.7290	0.3067	0.4685	0.6352	0.7153	0.1578	0.0004	0.2255	0.6352
	GAS-GLD	0.6047	0.7113	0.5765	0.4612	0.8089		0.7097	0.8485	0.1642	0.0011	0.2121	0.7625
	LMGAS-GHD	0.2497	0.3717	0.7759	0.3181	0.1084	0.1107	0.0000	0.0000	0.0000	0.0000	0.0000	0.0000
	LMGAS-GLD	0.3486	0.4459	0.7759	0.3629	0.1084	0.1107	0.0000	0.0000	0.0000	0.0000	0.0000	0.0000
	GARCH-GHD	0.6047	0.6177	0.6391	0.8422	0.8895	0.9305	0.7097	0.824	0.6508	0.2488	0.8461	0.7625
	GARCH-GLD	0.7540	0.8089	0.4612	0.5765	0.8895	0.9305	0.7583	0.8785	0.6096	0.0843	0.8461	0.7625
	FIAPARCH-GHD	0.1719	0.5295	0.4612	0.5765	0.6177	0.7767	0.3411	0.7764	0.7300	0.0843	0.824	0.7199
	FIAPARCH-GLD	0.6047	0.8089	0.2752	0.3605	0.6177	0.7767	0.7097	0.8785	0.5471	0.0478	0.824	0.7199

Table 7: Out-of-sample VaR backtesting for the returns

	Distribution	Unconditional Coverage						Conditional Coverage					
		Long position			Short position			Long position			Short position		
		1%	2.5%	5%	95%	97.5%	99%	1%	2.5%	5%	95%	97.5%	99%
Bitcoin	GAS-GHD	0.3093	0.3047	0.6631	0.7379	0.8153	0.5208	0.2115	0.5799	0.3004	0.3935	0.7923	0.7694
	GAS-GLD	0.6984	0.3047	0.4614	0.3014	0.8153	0.1584	0.2351	0.5799	0.3164	0.0177	0.7923	0.3594
	LMGAS-GHD	0.5186	0.1047	0.1014	0.241	0.8473	0.1575	0.0000	0.0000	0.0000	0.0000	0.0001	0.3579
	LMGAS-GLD	NA	0.0001	0.0001	NA	NA	NA	NA	0.0000	0.0000	NA	NA	NA
	GARCH-GHD	0.1876	0.3047	0.8959	0.7379	0.8153	0.3096	0.1712	0.5799	0.5511	0.3935	0.7923	0.5728
	GARCH-GLD	0.9543	0.3047	0.4614	0.4614	0.8153	0.1584	0.2065	0.5799	0.3164	0.3164	0.7923	0.3594
	FIAPARCH-GHD	0.6984	0.4104	0.8959	0.8585	0.8153	0.7754	0.2351	0.6883	0.9699	0.0032	0.7923	0.8923
	FIAPARCH-GLD	0.6984	0.4104	0.7766	0.3014	0.9871	0.5208	0.2351	0.6883	0.9238	0.0177	0.8533	0.7694
Ethereum	GAS-GHD	0.6984	0.4104	0.9815	0.6227	0.843	0.7754	0.8272	0.6883	0.5082	0.6681	0.5297	0.8923
	GAS-GLD	0.5208	0.4104	0.7766	0.6631	0.5368	0.7754	0.7694	0.6883	0.2798	0.4804	0.3985	0.8923
	LMGAS-GHD	0.5186	0.222	0.0026	0.0000	0.0000	0.0000	0.0017	0.0000	0.0000	0.0000	0.0000	0.0000
	LMGAS-GLD	0.7009	0.4135	0.902	0.0417	0.6458	0.0221	0.0106	0.0000	0.0000	0.0000	0.0026	0.0014
	GARCH-GHD	0.9543	0.4104	0.8959	0.7379	0.5368	0.5208	0.2065	0.3376	0.5511	0.6782	0.3985	0.7694
	GARCH-GLD	0.7754	0.3047	0.5575	0.9815	0.4104	0.5208	0.1572	0.3089	0.3129	0.6416	0.3234	0.7694
	FIAPARCH-GHD	0.7754	0.6823	0.8959	0.7766	0.8153	0.1584	0.8923	0.348	0.9699	0.5434	0.583	0.3594
	FIAPARCH-GLD	0.3096	0.2198	0.3014	0.4614	0.9871	0.1584	0.5728	0.2697	0.4838	0.3474	0.5695	0.3594
Litecoin	GAS-GHD	0.9543	0.8153	0.5575	0.3311	0.4973	0.7754	0.9099	0.7923	0.3129	0.1583	0.5227	0.8923
	GAS-GLD	0.7754	0.6823	0.5575	0.5152	0.8153	0.9543	0.8923	0.8452	0.3129	0.2673	0.583	0.9099
	LMGAS-GHD	0.0583	0.1557	0.014	0.1873	0.6864	0.957	0.0109	0.0154	0.009	0.0197	0.0774	0.0091
	LMGAS-GLD	0.3081	0.2524	0.0481	0.1014	0.2524	0.957	0.0442	0.0556	0.0023	0.0083	0.0622	0.0091
	GARCH-GHD	0.4796	0.4104	0.6631	0.9815	0.9871	0.6984	0.6779	0.6883	0.3004	0.5082	0.5695	0.8272
	GARCH-GLD	0.7754	0.6823	0.6631	0.9815	0.9871	0.6984	0.8923	0.8452	0.3004	0.5082	0.5695	0.8272
	FIAPARCH-GHD	0.5208	0.4973	0.9815	0.2566	0.9871	0.9543	0.7694	0.5227	0.8619	0.1154	0.5695	0.9099
	FIAPARCH-GLD	0.5208	0.9871	0.7766	0.4176	0.9871	0.9543	0.7694	0.5695	0.8883	0.2094	0.5695	0.9099
Ripple	GAS-GHD	0.7754	0.8153	0.5575	0.4176	0.4973	0.1876	0.1572	0.2432	0.1300	0.0000	0.0015	0.0179
	GAS-GLD	0.9543	0.8153	0.5575	0.4176	0.4973	0.1876	0.0091	0.2432	0.1300	0.0000	0.0015	0.0179
	LMGAS-GHD	0.0053	0.0035	0.0024	0.014	0.0000	0.0297	0.0203	0.0000	0.0000	0.0000	0.0000	0.0000
	LMGAS-GLD	0.0667	0.0035	0.0126	0.014	0.0000	0.0583	0.0049	0.0000	0.0000	0.0000	0.0000	0.0000
	GARCH-GHD	0.3093	0.9871	0.6631	0.8959	0.8153	0.5208	0.2115	0.0529	0.8636	0.5511	0.583	0.7694
	GARCH-GLD	0.4796	0.6496	0.8959	0.9815	0.6496	0.7754	0.6779	0.028	0.8879	0.5082	0.567	0.8923
	FIAPARCH-GHD	0.6984	0.4104	0.8959	0.9815	0.3644	0.9543	0.8272	0.6883	0.5511	0.0202	0.4478	0.9099
	FIAPARCH-GLD	0.6984	0.6823	0.8959	0.9815	0.2546	0.9543	0.8272	0.8452	0.5511	0.0202	0.3262	0.9099

Table 8: Volatility Forecast Evaluations for Bitcoin, Ethereum, Litecoin and Ripple.

	Distribution	RMSE	MAE	QLIKE	R^2 (log)
**Bitcoin**	GAS-GHD	0.0018	0.0007	512.1412	0.1650
	GAS-GLD	0.0019	0.0007	739.3266	0.1650
	LMGAS-GHD	0.0093	0.0036	NaN†	NaN†
	LMGAS-GLD	0.0091	0.003	55.672	0.000
	GARCH-GHD	0.0017	0.0011	-6.2708	0.0229
	GARCH-GLD	0.0017	0.0009	-6.3493	0.0229
	FIAPARCH-GHD	0.0021	0.0016	-6.012	0.0248
	FIAPARCH-GLD	0.0017	0.0009	-6.3072	0.0248
**Ethereum**	GAS-GHD	0.003	0.001	579.1387	0.1719
	GAS-GLD	0.003	0.001	545.5602	0.1719
	LMGAS-GHD	0.0056	0.0023	428.6561	0.000
	LMGAS-GLD	0.0056	0.0023	432.9261	0.000
	GARCH-GHD	0.0027	0.0012	-5.964	0.0294
	GARCH-GLD	0.0027	0.0013	-5.9614	0.0294
	FIAPARCH-GHD	0.0027	0.0013	-5.9403	0.031
	FIAPARCH-GLD	0.0027	0.0013	-5.9383	0.031
**Litecoin**	GAS-GHD	0.0041	0.0014	452.6532	0.0956
	GAS-GLD	0.0041	0.0014	441.4628	0.0956
	LMGAS-GHD	0.0064	0.0025	808.4961	0.000
	LMGAS-GLD	0.0064	0.0025	785.1924	0.000
	GARCH-GHD	0.0039	0.0017	-5.6662	0.0261
	GARCH-GLD	0.0039	0.0018	-5.6673	0.0261
	FIAPARCH-GHD	0.0039	0.0018	-5.6302	0.0071
	FIAPARCH-GLD	0.0039	0.0018	-5.6294	0.0071
**Ripple**	GAS-GHD	0.0111	0.0018	420.1552	0.0004
	GAS-GLD	0.0111	0.0018	523.8939	0.0004
	LMGAS-GHD	0.0204	0.0053	NaN†	NaN†
	LMGAS-GLD	0.0203	0.0051	43.1707	0.000
	GARCH-GHD	0.0113	0.0023	-3.877	0.0364
	GARCH-GLD	0.0127	0.0033	-4.7613	0.0364
	FIAPARCH-GHD	0.0124	0.0031	-5.4077	0.0480
	FIAPARCH-GLD	0.0117	0.0026	-5.3934	0.0480

† QLIKE not defined due to instability in conditional variance estimates.
